# The Spectre of Too Many Species

**DOI:** 10.1093/sysbio/syy051

**Published:** 2018-07-05

**Authors:** Adam D Leaché, Tianqi Zhu, Bruce Rannala, Ziheng Yang

**Affiliations:** 1 Department of Biology & Burke Museum of Natural History and Culture, University of Washington, Seattle, USA; 2 National Center for Mathematics and Interdisciplinary Sciences, Academy of Mathematics and Systems Science, Chinese Academy of Sciences, China; 3 Key Laboratory of Random Complex Structures and Data Science, Academy of Mathematics and Systems Science, Chinese Academy of Sciences, China; 4 Department of Evolution and Ecology, University of California Davis, One Shields Avenue, Davis, USA; 5 Department of Genetics, University College London, London, UK; 6 Radcliffe Institute for Advanced Studies, Harvard University, Cambridge, USA

**Keywords:** bpp, multispecies coalescent, Species delimitation, taxonomy

## Abstract

Recent simulation studies examining the performance of Bayesian species delimitation as implemented in the bpp program have suggested that bpp may detect population splits but not species divergences and that it tends to over-split when data of many loci are analyzed. Here, we confirm these results and provide the mathematical justifications. We point out that the distinction between population and species splits made in the protracted speciation model (PSM) has no influence on the generation of gene trees and sequence data, which explains why no method can use such data to distinguish between population splits and speciation. We suggest that the PSM is unrealistic as its mechanism for assigning species status assumes instantaneous speciation, contradicting prevailing taxonomic practice. We confirm the suggestion, based on simulation, that in the case of speciation with gene flow, Bayesian model selection as implemented in bpp tends to detect population splits when the amount of data (the number of loci) increases. We discuss the use of a recently proposed empirical genealogical divergence index (*gdi*) for species delimitation and illustrate that parameter estimates produced by a full likelihood analysis as implemented in bpp provide much more reliable inference under the *gdi* than the approximate method phrapl. We distinguish between Bayesian model selection and parameter estimation and suggest that the model selection approach is useful for identifying sympatric cryptic species, while the parameter estimation approach may be used to implement empirical criteria for determining species status among allopatric populations.

In the past decade, the multispecies coalescent (MSC) model (Rannala and Yang 2003) has emerged as an important framework for statistical analysis of genomic sequence data from closely related species. Under the model, different genomic regions (called loci) may have different genealogical histories due to coalescent processes occurring in the extinct ancestral species. The MSC thus naturally accommodates gene tree heterogeneity across the genome. Likelihood-based inference under the MSC averages over the gene trees for multiple loci, achieved through either numerical integration (Yang 2002; Zhu and Yang 2012) or Bayesian Markov chain Monte Carlo (MCMC) (Edwards 2009; Heled and Drummond 2010; Yang and Rannala 2010, 2014). Averaging over gene trees incurs a heavy computational burden but has the benefit of accommodating phylogenetic uncertainty at individual loci, which is important when the species are closely related and the sequence alignment at each locus has low phylogenetic information content (Xu and Yang 2016). Given the species phylogeny, the MSC can be used to estimate important parameters concerning species divergences such as the population sizes of modern and ancestral species, species divergence times, and past migration patterns and rates (Takahata et al. 1995; Burgess and Yang 2008; Hey 2010; Mailund et al. 2012). The MSC also provides the appropriate inference framework for estimating species phylogenies, while accommodating gene tree heterogeneity caused by deep coalescence and incomplete lineage sorting (Maddison 1997; Nichols 2001; Edwards 2009; Heled and Drummond 2010; Yang and Rannala 2014). It has been applied to species identification (assignment) and found to achieve better statistical performance than DNA bar-coding based on a simple distance threshold (Yang and Rannala 2017). The MSC has also been used to address the problem of species discovery (or delimitation) (Yang and Rannala 2010, 2014). Different species delimitation models are formulated as competing statistical models and inferred from the genetic data through Bayesian model selection (i.e., through calculation of posterior model probabilities). Species delimitation is a complex issue, however, partly because there is no universally accepted definition of species (Mallet 2013).

Two recent studies (Jackson et al. 2017; Sukumaran and Knowles 2017) used computer simulation to evaluate the performance of Bayesian species delimitation as implemented in the software package bpp (Bayesian Phylogenetics and Phylogeography) (Yang and Rannala 2010; Rannala and Yang 2013). Both studies concluded that bpp may over-split, capturing population splits rather than species divergences. Sukumaran and Knowles (2017) simulated species phylogenies, gene trees, and sequence data under the protracted speciation model (PSM) (Rosenblum et al. 2012; Etienne et al. 2014), which distinguishes between populations (incipient species) and species. They concluded that in some cases bpp delimited population structure rather than species. Jackson et al. (2017) simulated sequence data under the MSC model on a given species tree, and then used a heuristic genealogical divergence index (*gdi*) to define species status. They found that their simulation-based heuristic method phrapl was more successful in inferring species status than bpp, which tended to split subdivided populations into species even in the face of high gene flow.

Here, we examine the conditions of the simulations of Sukumaran and Knowles (2017) and Jackson et al. (2017) to evaluate the performance of bpp. Two features of the simulation of Sukumaran and Knowles (2017) are noteworthy. First, the species conversion process is superimposed on the population branching process and is Markovian (memoryless) so the rate of species conversion (from incipient species to species) is fixed and independent of the duration of genetic isolation between incipient species. Moreover, the PSM distinguishes between populations and species but the species status of lineages is ignored when the gene trees and sequence data are generated under the MSC model for subsequent analysis using bpp. Second, the assignment of species status in the PSM does not appear to be consistent with current taxonomic practices or with most models of speciation.

In Jackson et al. (2017), a heuristic criterion was used to define species and that definition was used in phrapl but not in bpp when both programs were used to infer species status. We perform a fair comparison in which the same heuristic species definition is used in both phrapl and bpp analyses. We demonstrate that even though bpp ignores gene flow and is based on the simplistic JC mutation model (Jukes and Cantor 1969), it provides more accurate parameter estimates and inference of species status than phrapl when both programs use the same heuristic definition of species. We discuss the asymptotic behavior of Bayesian species delimitation through model selection as the number of loci increases.

## Protracted Speciation?

A defining feature of the simulation by Sukumaran and Knowles (2017) under the PSM is that the conversion event that transforms a population (an incipient species) into a species (a true species) is independent of the process of genetic divergence among populations and of the generation of gene trees and sequence data. The PSM distinguishes between populations and species but when the population tree is used to simulate gene trees and sequences no such distinction is made. The simulation may be considered an attempt to mimic the use of the neutral genome or non-coding DNA to delineate species boundaries, but the procedure makes it clear that the simulated sequence data do not contain information concerning species status. This is a consequence of the likelihood principle in statistics, which states that all information about the competing models and model parameters is contained in the likelihood function, the probability of the data given the model and parameters (O’Hagan and Forster, 2004, pp. 61–64). If two models make the same probabilistic predictions about the observable data and thus have identical likelihoods for all possible data outcomes, the models are not identifiable and the data cannot be used to distinguish them.

The PSM used in the simulation of Sukumaran and Knowles (2017) is a simplified version in which the “species initiation rate” (rate at which incipient species arise) is identical for incipient and true species, as is the species extinction rate. Thus the model has three parameters, species initiation rate $\lambda$, species extinction rate $\mu$, and species conversion rate $\beta$. Because the rates $\lambda$ and $\mu$ do not depend on the status of the population (incipient species or true species) it is straightforward to study the statistical properties of this model by first determining the probability density of the population tree under a conventional birth–death process and then superimposing the process of species conversion on the population tree.

**Figure 1.**
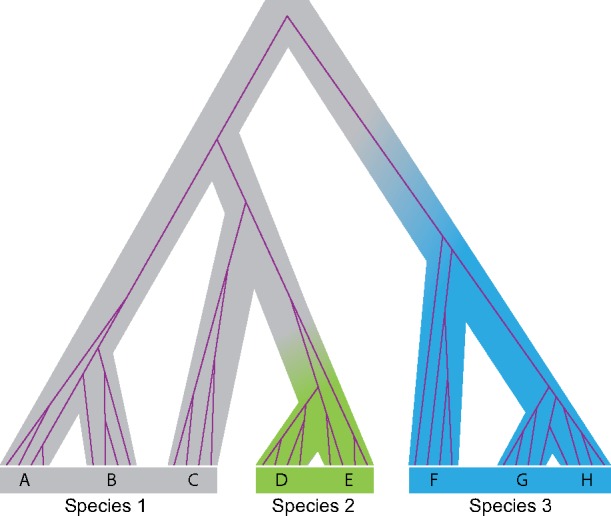
Figure 1 of Sukumaran and Knowles (2017) redrawn to illustrate the simulation of species (indicated by tip labels) under the protracted speciation model. The species tree is shown with one embedded gene tree (purple); a species conversion event happens when a branch on the species tree changes color.

Let $S$ be the population tree topology and $\boldsymbol{\tau}$ be the set of divergence times (Fig. 1). Let $\boldsymbol{ \theta }$ be the set of population size parameters, with $\theta = 4N\mu$ where $N$ is the effective population size and $\mu$ is the mutation rate per generation per site. Both parameters $\tau$ and $\theta$ are measured in the expected number of mutations per site. Let $\Lambda$ be the species delimitation (or a representation of coloring scheme in Fig. 1). Let the sequence data at the $L$ loci be $\mathbf{X} = \{ X_i \}$, $i = 1, \cdots, L$, and let the gene trees be $\mathbf{G} = \{ G_i \}$. Bayesian species delimitation under the PSM should then involve a slight change to the formulation of Yang and Rannala (2010). The posterior probability distribution of species delimitation, species/population tree as well as the parameters in the MSC on the population tree ($\theta$s and $\tau$s) is then

(1) \begin{eqnarray*} f(\Lambda, S, \boldsymbol{ \tau }, \boldsymbol{ \theta } | \mathbf{X}) & \propto & f(\Lambda, S, \boldsymbol{ \tau } | \lambda, \mu, \beta) \times f(\boldsymbol{ \theta } |S) \nonumber \\ & \times & \prod_{i=1}^L \int_{G_i} f(G_i|S, \boldsymbol{\tau}, \boldsymbol{\theta}) f(X_i | G_i) \, \textrm{d} G_i. \end{eqnarray*}

Here, the MSC density for the gene tree topology and coalescent times, $f(G_i|S, \boldsymbol{\tau}, \boldsymbol{\theta})$, is given by Rannala and Yang (2003), the probability of the sequence alignment at locus $i$ (known as the phylogenetic likelihood), $f(X_i | G_i)$, is given by Felsenstein (1981).

The joint prior for the population tree and species delimitation factors into two terms:

(2) \begin{equation*} f(\Lambda, S, \boldsymbol{ \tau } | \lambda, \mu, \beta) = f(S, \boldsymbol{ \tau } | \lambda, \mu) f(\Lambda| S, \boldsymbol{ \tau }, \beta), \end{equation*}

where $f(S, \boldsymbol{ \tau } | \lambda, \mu)$ is the density for the population tree and divergence times given by the birth–death process (e.g., Rannala and Yang, 1996), while $f(\Lambda | S, \boldsymbol{ \tau }, \beta)$ is the probability of the species delimitation (species conversions) given the population tree, which is specified by the Poisson process, with constant rate $\beta$, of species conversions running along the branches of the population tree.

For example, given the population tree for eight populations of Figure 1, the probability of the delimitation in Figure 1 (i.e., the probability of the coloring scheme with the two conversion events and with the three true species) is

(3) \begin{eqnarray*} &&f(\Lambda| S, \boldsymbol{ \tau }, \beta)\nonumber\\ && = \beta^2 \mathrm{e}^{-\beta [2\tau_{ABCDEFGH} + \tau_{ABCDE} + \tau_{AB} + \tau_{CDE} + \tau_{DE} + \tau_{FGH} + \tau_{GH} ]},\quad \end{eqnarray*}

where $\tau_{AB}$ is the age of the $AB$ ancestor, and so on. Note that the term in the square bracket is the total time length of the population tree.

From this formulation, it is clear that the impact of the PSM is to change the prior, since the sequence likelihood and the MSC density for the gene trees are unchanged. The fact that the species conversion process is conditionally independent of the population tree means that the genetic data do not allow species to be delimited without assuming the rate $\beta$. It then follows that the posterior probabilities for the species delimitation models will be extremely sensitive to the conversion rate $\beta$ or its prior.

### The Protracted Speciation Model Assumes Instantaneous Speciation

The PSM assumed in the simulation of Sukumaran and Knowles (2017) has several extreme properties, making it an unrealistic model for most speciation processes in nature. The model posits an exaggerated form of punctuated equilibrium—exponentially distributed periods of stasis followed by an instantaneous conversion to a new species. At the conversion event, the new population and the parental population (which are only one generation apart) are deemed distinct species. Even though Sukumaran and Knowles (2017) used the PSM to simulate speciation as an extended process rather than an event, PSM assumes instantaneous speciation or conversion of an incipient species into true species in one generation. Few species appear to have originated in this way. An alternative “gradualist” model would treat morphological characters involved in species classification as quantitative traits that evolve according to a diffusion model determined by the effects of underlying mutational changes and genetic drift of allele frequencies. Two populations are recognized as different species if the difference in mean trait values exceeds some threshold, which reflects the biologist’s perception of what species are and how morphologically different distinct species should be. Under such a model there will be a strong covariance between genetic isolation, population divergence time, and species status. This gradualist model is another extreme and a more realistic model may include a mixture of morphological “jumps” as well as “diffusions” (see Discussion section).

The way in which the PSM assigns species status is also problematic, contradicting prevailing taxonomic practices. In Figure 1 of Sukumaran and Knowles (2017), the different colors on branches signify distinct species produced by conversion events under the PSM (Fig. 1). It is possible for the model to generate species near the tips of the species tree, say, $<10$ generations ago. However, taxonomists would not recognize recent divergences of only a few generations as valid speciation events. Instead, speciation is a consequence of an extended process of genetic isolation, and species status is assigned retrospectively based on empirical measures of morphological and/or genetic divergence. It may not be possible to simulate species forward in time because the criterion of the systematist depends on the level of divergence between populations and this is only known after the simulation of population splits is completed.

## Asymptotic Behavior of Bayesian Comparison of Species Delimitation Models

Jackson et al. (2017) simulated data under the MSC model with migration (Hey, 2010, the so-called isolation-with-migration or IM model) for two species/populations and analyzed them using bpp to calculate the posterior probabilities for the one-species and two-species models. They observed that the posterior probability for the two-species model increases when the number of loci increases. Here, we investigate the asymptotic behavior of Bayesian posterior model probabilities and confirm that this is the expected behavior of Bayesian model selection and of the program.

### Choosing Among Wrong Models

The asymptotic dynamics of Bayesian model selection depends on how wrong the two competing models are relative to the true data-generating model (Yang and Zhu 2018). Here, we consider independent and identically distributed (i.i.d.) models only, under which the data points $x_i\ (i = 1, \cdots, L)$ are i.i.d., with $x_i \sim q(x_i)$. Let $X = \{x_i\}$. The distance from any model $p(x | \phi)$ with parameters $\phi$ to the true model $q$ is measured by the Kullback–Leibler (KL) divergence

(4) \begin{equation*} \label{eq:KL} D = \int q(x) \log \frac{q(x)}{p(x|\phi^*)} \, \mathrm{d}x, \end{equation*}

where $\phi^*$ is the limiting maximum likelihood estimate (MLE) of $\phi$ under the model when the data size $L \rightarrow \infty$, and is known as the best-fitting parameter value under the model (White 1982). The KL divergence $D = 0$ if the model encompasses the true model (or, in other words, is true), and $D > 0$ if the model is wrong.

**Figure 2.**
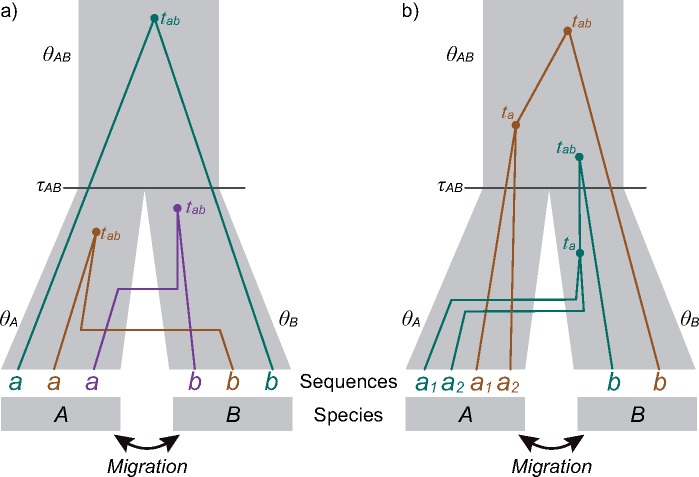
a) A species tree for two species ($A$ and $B$) and three gene trees for two sequences ($a$ and $b$), used to illustrate the asymptotics of Bayesian model selection. The coalescence between the two sequences occurs before species divergence in the brown and purple gene trees (with $t < \tau$) and after in the green gene tree (with $t > \tau$). b) A species tree for two species ($A$ and $B$) and two gene trees for three sequences ($a_1$ and $a_2$ from species $A$ and $b$ from species $B$), used to illustrate the computation of the *gdi*. Both gene trees have the same topology $G_1 = ((a_1, a_2), b)$, but the coalescence between $a_1$ and $a_2$ occurs before species divergence (in species $B$) in the green tree (with $t_a < \tau_{AB}$) and after in the brown tree (with $t_a > \tau_{AB}$).

Here the true model $q$ is the MSC model with migration (the IM model). Under the model, the gene trees and sequence alignments are i.i.d. among loci, so that the datasize is the number of loci ($L$). Currently, bpp does not accommodate migration or introgression and implements the complete isolation model only. The two models under comparison are then the one-species model $(H_1)$ with a single population-size parameter $\phi_1 = \{ \theta \} $ and the two-species model $(H_2)$ with parameters $\phi_2 = \{\tau, \theta_A, \theta_B, \theta_{AB} \}$, where $\tau$ (for $\tau_{AB}$) is the divergence time between the two species, and the $\theta$s are the population size parameters for the two modern species $A$ and $B$ and for the ancestral species $AB$, with $\theta = 4N\mu$ (Fig. 2a). Both $\theta$ and $\tau$ are measured in the expected number of mutations per site. As the true model involves migration, both models $H_1$ and $H_2$ are wrong, with $D_1 > 0, D_2 > 0$. Note that $H_1$ is a special case of $H_2$ since the two models are equivalent when $\tau = 0$ in $H_2$, in which case parameters $\theta_A$ and $\theta_B$ in $H_2$ are unidentifiable. The dynamics of the posterior probabilities for $H_1$ and $H_2$ depends on whether $H_1$ and $H_2$ are equally wrong (in which case $D_1 = D_2 > 0$) or $H_2$ is less wrong than $H_1$ (with $D_1 > D_2 > 0$), or equivalently on whether the best fitting parameter value for $\tau$ in $H_2$ is $\tau^* = 0$ or $> 0$. If $\tau^* = 0$, the two models will be equally wrong, and they are also unidentifiable in the limit of infinite data. Then $H_1$, with fewer parameters, dominates, with its posterior probability approaching 100% when the number of loci $L$ increases. In contrast, if $\tau^* > 0$, $H_2$ is less wrong than $H_1$, and $H_2$ will dominate. While an analytical proof is not available, we analyze increasingly larger data sets to examine the asymptotic behavior of the MLEs numerically. Our calculations suggest that the second case applies: when the true model is the MSC model for two populations with migration, the two-species isolation model $H_2$ is less wrong than the one-species model $H_1$ and dominates in the posterior when the number of loci increases.

As an example, we simulate large data sets with many loci, each of 500 sites, under the symmetrical IM model for two species with $\tau = 0.01$ for the species divergence and $\theta_A = \theta_B = \theta_{AB} = \theta = 0.01$ for all populations, and with migration rates between the two populations to be $M_{AB} = M_{BA} = M = Nm = 10$ immigrants per generation (Fig. 2a). In this article, the (scaled) migration rate is defined as $M_{ij} = N_j m_{ij}$, the expected number of immigrants in population $j$ from population $i$ per generation, with $m_{ij}$ to be the proportion of immigrants in population $j$. The MCcoal program, in the bpp package, was used to generate gene trees and sequence alignments under the JC model (Jukes and Cantor 1969). Each locus has two sequences, $a$ and $b$, from species $A$ and $B$, respectively. At those parameter values, sequences $a$ and $b$ coalesce before species divergence (with $t < \tau$, as in the brown and purple gene trees of Fig. 2a) at 62.75% of loci, which is very similar to the probability for $t < \tau$ (63.21%) if the two sequences are from the same population.

The data are then analyzed using the 3S program to obtain the MLEs for the two parameters ($\theta_{AB}$ and $\tau$) under the two-species MSC model with no migration ($H_2$) (Yang 2002; Dalquen et al. 2017). The estimate of $\theta_{AB}$ is 0.0158. The MLE $\hat{\tau}$ ranged from 0.00033$-$0.00036 over ten replicates for $L = 10^5$ to over 0.000329$-$0.000348 for $L = 2 \times 10^5$. Based on the stability of the estimates among the replicate data sets and between the large values of $L$, we suggest that at the limit of infinitely many loci, the best-fitting parameter value is $\tau^* = 0.00034$. We note that the best-fitting parameter value depends on the configuration of the data such as the number of sequences per locus and the number of sites, as well as the parameters of the MSC model with migration ($\tau$s, $\theta$s, and $M$’s). If the sequence length is 250 sites instead of 500, we obtain $\tau^* \sim 0.00062$ instead of 0.00034. Those results provide numerical evidence that at the limit of infinite data, $\tau^* > 0$, so that the two-species model will dominate the posterior, even though the migration rates are so high between the two populations that they should be considered one species by any species definition.

### The Impact of Migration or Gene Flow

Note that if Bayesian model selection is conducted under the IM model, incorporating migration, the two-species model with migration will be correct (with $D_2 = 0$), while the one-species model will be wrong (with $D_1 > 0$). Then the two-species model will dominate with the posterior probability approaching 100% as the number of loci increases. This is the case even if the migration rate $M = Nm$ is very large (but finite). Thus if we use Bayesian model selection to infer species status (treating a population split as a speciation event) then incorporating migration into the MSC model will not correct the problem of over-splitting.

In conclusion, the concern that Bayesian model selection as implemented in bpp may over-split and recognize too many species in subdivided populations with ongoing gene flow is legitimate. Over-splitting may be of particular concern when hundreds or thousands of loci are analyzed. If two populations are truly panmictic, the model with fewer parameters will be favored, and the populations will be correctly lumped into one species. However, if there is partial subdivision (even with relatively high levels of gene flow) the method will prefer the two-species model asymptotically as the number of loci increases. One possible solution is to include a model with gene flow and use model selection to choose among 3 models: (i) a single population; (ii) two completely isolated populations; and (iii) two populations with gene flow. A choice of model 1 strongly suggests a single species; a choice of model 2 suggests two species but a final decision should be based on a consideration of the population divergence time and other relevant information (morphology, etc); a choice of model 3 allows either one species or two, depending on considerations such as the degree of gene flow, distinctness of morphology, and so on.

## Heuristic Species Delimitation

Jackson et al. (2017) suggested a heuristic criterion for species delimitation based on a genealogical divergence index (*gdi*) between populations that can be calculated using estimates of parameters under the MSC model with migration ($\tau$, $\theta$, and $M$). Suppose one samples two sequences ($a_1$ and $a_2$) from population $A$ and one sequence ($b$) from population $B$ (see Fig. 2b). Let the probability that the two sequences from population $A$ coalesce first, so that the gene tree is $G_1 = ((a_1, a_2), b)$, be

(5) \begin{equation*} \label{eq:PG1} P_1 = \mathbb{P}(G_1|\tau, \theta_A, \theta_B, \theta_{AB}, M_{AB}, M_{BA}). \end{equation*}

Obviously $P_1$ ranges from $\frac{1}{3}$ (when the three sequences are interchangeable, as in the case of $M_{AB} = M_{BA} = \infty$) to 1. Jackson et al. (2017) rescaled $P_1$ so that the genealogical divergence index,

(6) \begin{equation*} \label{eq:gdi} \textit{gdi} = (3 \times P_1 - 1)/2, \end{equation*}

ranges from 0 to 1 when $P_1$ goes from $\frac{1}{3}$ to 1. In the special case of no migration (with $ M_{AB} = M_{BA} = 0$), we have $ P_1 = 1 - \frac{2}{3} \mathrm{e}^{-2 \tau / \theta_A} $ and

(7) \begin{equation*} \label{eq:PG1_M0} gdi = 1 - \mathrm{e}^{-2\tau / \theta_A}, \end{equation*}

where $2\tau/\theta_A$ is the population divergence time in coalescent units (with one coalescent time unit to be $2N_A$ generations) and $\mathrm{e}^{-2\tau/\theta_A}$ is the probability that the two sequences from population $A$ ($a_1$ and $a_2$) do not coalesce before reaching species divergence ($\tau$) when we trace the genealogy backwards in time.

### The gdi Heuristic for Species Identification

Jackson et al. (2017) calculated the *gdi* as defined in equations 5 and 6 by simulating 10,000 gene trees under the MSC model with migration. Here, we provide its analytical computation, using the Markov chain characterization of the coalescent process with migration (Hobolth et al. 2011; Zhu and Yang 2012; Dalquen et al. 2017). For two populations ($A$ and $B$) with gene flow and three sequences ($a_1$, $a_2$, and $b$), the genealogical process of coalescent and migration when one traces the history of the sample backwards in time can be described by a Markov chain with 21 states. The state of the chain is specified by the number of sequences remaining in the sample and the populations in which they reside, or by the population IDs ($A$ and $B$) and the sequence IDs ($a_1, a_2, b$, etc.). For example, the state $A_{a_1}A_{a_2}B_b$ means that the three sequences $a_1, a_2$, and $b$ are in populations $A$, $A$, and $B$, respectively. We also write this as “$AAB$”. This is the initial state. State $A_{a_1a_2}B_b$, abbreviated “$AB_b$”, means that two sequences remain in the sample, with the ancestor of sequences $a_1$ and $a_2$ in population $A$ and sequence $b$ in population $B$.

The transition rate matrix of the Markov chain $Q = \{q_{ij}\}$ is given in Table 1. The transition probability matrix over time $t$ is then $P(t) = \{p_{ij}(t)\} = \mathrm{e}^{Qt}$, where $p_{ij}(t)$ is the probability that the Markov chain is in state $j$ at time $t$ in the past given that it is in state $i$ at time 0 (the present time). Suppose $Q$ has the spectral decomposition

(8) \begin{equation*} q_{ij} = \sum_{k=1}^{21} u_{ik} v_{kj} \lambda_k , \end{equation*}

**Table 1.** Rate matrix for Markov chain describing transitions between states in multispecies coalescent with migration model with two populations ($A$ and $B$) and three sequences ($a_1$, $a_2$, and $b$)

	$AAA$	$AAB$	$ABA$	$ABB$	$BAA$	$BAB$	$BBA$	$BBB$	$A_{a_1}A$	$A_{a_2}A$	$A_{b}A$	$B_{a_1}B$	$B_{a_2}B$	$B_{b}B$	$A_{a_1}B$	$A_{a_2}B$	$A_{b}B$	$AB_{a_1}$	$AB_{a_2}$	$AB_{b}$	$A|B$
$AAA$	$\cdot$	$w_{BA}$	$w_{BA}$		$w_{BA}$				$c_A$	$c_A$	$c_A$										
$AAB$	$w_{AB}$	$\cdot$		$w_{BA}$		$w_{BA}$														$c_A$	
$ABA$	$w_{AB}$		$\cdot$	$w_{BA}$			$w_{BA}$												$c_A$		
$ABB$		$w_{AB}$	$w_{AB}$	$\cdot$				$w_{BA}$							$c_B$						
$BAA$	$w_{AB}$				$\cdot$	$w_{BA}$	$w_{BA}$											$c_A$			
$BAB$		$w_{AB}$			$w_{AB}$	$\cdot$		$w_{BA}$								$c_B$					
$BBA$			$w_{AB}$		$w_{AB}$		$\cdot$	$w_{BA}$									$c_B$				
$BBB$				$w_{AB}$		$w_{AB}$	$w_{AB}$	$\cdot$				$c_B$	$c_B$	$c_B$							
$A_{a_1}A$									$\cdot$						$w_{BA}$			$w_{BA}$			$c_A$
$A_{a_2}A$										$\cdot$						$w_{BA}$			$w_{BA}$		$c_A$
$A_bA$											$\cdot$						$w_{BA}$			$w_{BA}$	$c_A$
$B_{a_1}B$												$\cdot$			$w_{AB}$			$w_{AB}$			$c_B$
$B_{a_2}B$													$\cdot$			$w_{AB}$			$w_{AB}$		$c_B$
$B_bB$														$\cdot$			$w_{AB}$			$w_{AB}$	$c_B$
$A_{a_1}B$									$w_{AB}$			$w_{BA}$			$\cdot$						
$A_{a_2}B$										$w_{AB}$			$w_{BA}$			$\cdot$					
$A_bB$											$w_{AB}$			$w_{BA}$			$\cdot$				
$AB_{a_1}$									$w_{AB}$			$w_{BA}$						$\cdot$			
$AB_{a_2}$										$w_{AB}$			$w_{BA}$						$\cdot$		
$AB_b$											$w_{AB}$			$w_{BA}$						$\cdot$	
$A|B$																					$\cdot$

*Note:* $w_{AB} = 4M_{AB}/\theta_B = m_{AB}/\mu$ and $w_{BA} = 4M_{BA}/\theta_A = m_{BA}/\mu$ are mutation-scaled migration rates, and $c_A = 2/\theta_A$ and $c_B = 2/\theta_B$ are the coalescent rates. The state of the chain is given by the population IDs ($A$ or $B$) and sequence IDs (such as $a_1$, $a_2$, $a_1a_2$). For example the initial state $A_{a_1}A_{a_2}B_b$ means that the three sequences $a_1, a_2$, and $b$ are from populations $A$, $A$, and $B$, respectively. States with three sequences are abbreviated, with the three sequences assumed to be in the order $a_1, a_2, b$ so that the sequence IDs are suppressed. Thus $A_{a_1}A_{a_2}B_b$ is ‘$AAB$’. State $A_{a_1a_2}B_b$ means that two sequences remain in the sample, with the ancestor of sequences $a_1$ and $a_2$ is in population $A$ while sequence $b$ is in population $B$. This is abbreviated ‘$AB_b$’, with the sequence ID ‘$a_1a_2$’ suppressed. ‘$A|B$’ is an absorbing state in which only one sequence remains in the sample, in either $A$ or $B$, after two coalescent events have occurred.

where $\lambda_k$ are the eigenvalues of $Q$, columns in $U = \{u_{ij}\}$ are the corresponding right eigenvectors, and rows in $V = \{v_{ij}\} = U^{-1}$ are the left eigenvectors. Then

(9) \begin{equation*} \label{eq:Pt} p_{ij}(t) = \sum_{k=1}^{21} u_{ik} v_{kj} \mathrm{e}^{\lambda_k t}. \end{equation*}

Gene tree $G_1 = ((a_1, a_2), b)$ can be generated in two ways. The first is for sequences $a_1$ and $a_2$ to coalesce before reaching the ancestral population, with $t < \tau$ (as in the green gene tree of Fig. 2b). Sequence $b$ then joins the ancestor of sequences $a_1$ and $a_2$ either before species divergence at $\tau$, in which case the root of the gene tree is younger than species divergence, or after, in which case the root of the gene tree is older than $\tau$ (the latter case is illustrated in the green gene tree of Fig. 2b).

The probability density that sequences $a_1$ and $a_2$ coalesce at time $t < \tau$ is given by

(10) \begin{eqnarray*} \label{eq:ft} f(t) &=& [p_{AAB, AAA}(t) + p_{AAB, AAB}(t)] \times \frac{2}{\theta_A} \nonumber \\ &+& [p_{AAB, BBA}(t) + p_{AAB, BBB}(t)] \times \frac{2}{\theta_B}, \ \ \ t < \tau. \end{eqnarray*}

This is a sum of two terms, corresponding to the first coalescent (between sequences $a_1$ and $a_2$) occurring in populations $A$ and $B$, respectively. The first term is the probability, $p_{AAB, AAA}(t) + p_{AAB, AAB}(t)$, that sequences $a_1$ and $a_2$ are in population $A$ right before time $t$, times the rate for them to coalesce $(\frac{2}{\theta_A})$. Similarly, the second term is the probability density that sequences $a_1$ and $a_2$ coalesce at time $t$ in population $B$ (Fig. 2b, green gene tree).

The second way of generating gene tree $G_1$ is for sequences $a_1$ and $a_2$ to coalesce after population divergence, with $t > \tau$ (as in the brown gene tree of Fig. 2b). This occurs with probability $P_{AAB, S_3}(\tau) \times \frac{1}{3}$, where $S_3 = \{AAA, AAB, ABA, ABB, BAA, BAB, BBA, BBB\}$ is the set of states with three sequences, and $P_{AAB, S_3}(\tau)$ is the probability that no coalescent event occurs during the time interval $(0, \tau)$. In this scenario, the gene tree root must be older than $\tau$.

Thus combining the two possibilities for generating gene tree $G_1$, we have

(11) \begin{equation*} \label{eq:P0} P_1 = \int_0^\tau f(t) \; \mathrm{d}t + P_{AAB, \, S_3}(\tau) \times \frac{1}{3}, \end{equation*}

where $f(t)$ is given in equation 10. To calculate the integral in equation 11, note that from equation 9,

(12) \begin{equation*} \int_0^\tau p_{ij}(t) \mathrm{d} t = \sum_{k=1}^{21} u_{ik} v_{kj} \frac{1}{\lambda_k} \left(\mathrm{e}^{\lambda_k \tau} - 1\right). \end{equation*}

**Figure 3.**
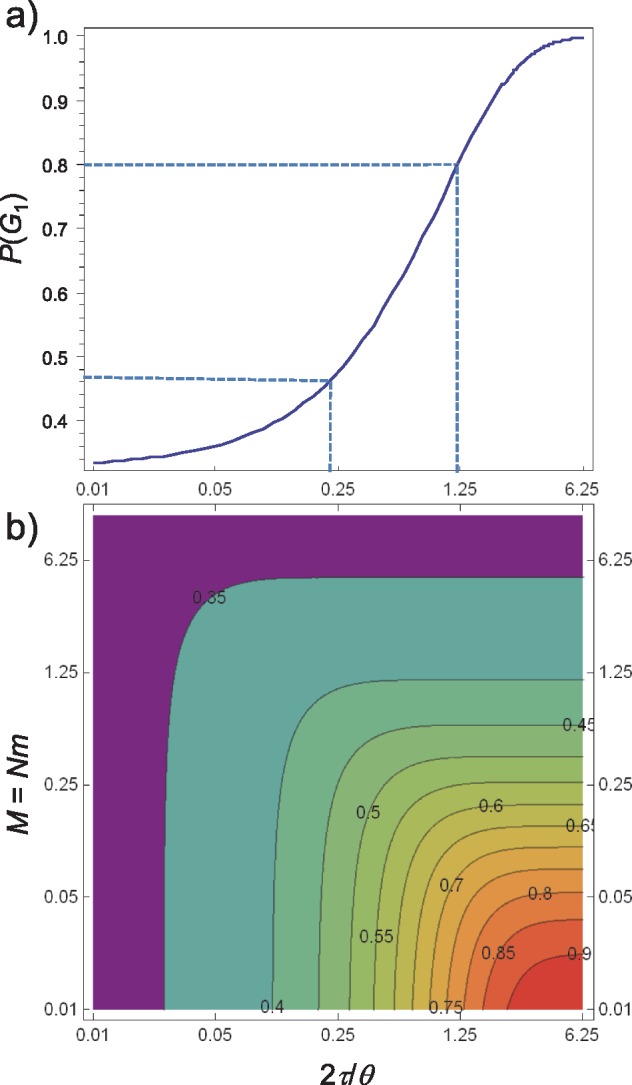
Probability $\mathbb{P}(G_1)$ of gene tree $G_1 = ((a_1, a_2), b)$, plotted (a) as a function of population divergence in coalescent units ($2\tau/\theta$) in a pure isolation model for two populations without gene flow and (b) as a function of population divergence in coalescent units ($2\tau/\theta$) and scaled migration rate $M = Nm$. According to Jackson et al. (2017), the lower and upper limits of $P_1$ for species delimitation are 0.47 and 0.8.

We calculated $P_1$ under the symmetrical migration model with $\theta_A = \theta_B = \theta$ and $M_{AB} = M_{BA} = M = Nm$. Figure 3b shows $P_1$ plotted against $2\tau/\theta$ (population divergence time in coalescent units) and $M$ under the symmetrical migration model. This is a more accurate calculation than Figure 6 of Jackson et al. (2017), which was based on simulating gene trees, even though the two approaches are equivalent if a huge number of replicates is used in the simulation.

Based on the meta-analysis of Pinho and Hey (2010), Jackson et al. (2017) suggested the rule of thumb that *gdi* values $<0.2$ suggest a single species and *gdi* values $>0.7$ suggest distinct species, while *gdi* values within the range indicate ambiguous delimitation. The limits of 0.2 and 0.7 for *gdi* correspond to 0.47 and 0.8 for $P_1$, and in the case of no migration, to 0.22 and 1.20 for the population divergence in coalescent units ($2\tau/\theta$) (Fig. 3a).

## Subjectively Defined Species

Jackson et al. (2017) simulated data under the MSC model with migration for two populations and analyzed the data using phrapl and bpp. While the true model used in the simulation always had two populations, the *gdi* was used to define species status. This criterion was used in the phrapl analysis of the simulated data to infer species status, but not in bpp. It was then found that phrapl out-performed bpp (Jackson et al. 2017, Fig. 4), and that bpp tended to over-split, identifying too many species.

### Comparison Between phrapl and bpp

Both bpp and phrapl can estimate the parameters of the MSC model, although phrapl accommodates gene flow, while bpp in its current implementation assumes no gene flow. Here, we apply the *gdi* definition of species status in bpp, so that the same criterion is used by bpp and phrapl. A simple approach is to use the posterior means of the parameters under the MSC generated by bpp to calculate the *gdi* (equation 7). We use this method here. A more sophisticated approach, which we use later in the analysis of the empirical data sets, is to generate a posterior distribution of *gdi* using the sample of parameters taken during the MCMC.

We thus repeated the simulation of Jackson et al. (2017, Fig. 4), applying *gdi* to bpp parameter estimates. The true species tree is $((A, B), C)$, with six sets of species divergence time parameters, with $\tau_{AB} = 0.05\theta, 0.125\theta, 0.25\theta, 1\theta, 2\theta$, $4\theta$, and $\tau_{ABC} = 2.5\theta, 2.5\theta, 2.5\theta, 2.5\theta, 5\theta, 10\theta$, with $\theta = 0.005$. Note that $\tau_{ABC}$ is much larger than $\tau_{AB}$, so that species $C$ is a distant outgroup, and the focus is on whether populations $A$ and $B$ are one or two species. Migration is assumed to occur between $A$ and $B$, with $4Nm = 0, 0.5, 2$, and $5$, where $Nm$ is the number of immigrants per generation. The sequence data were simulated under the HKY model (Hasegawa et al. 1985), with base frequencies 0.3, 0.2, 0.3, and 0.2 (for T, C, A, and G) and transition/transversion rate ratio $\kappa = 3$. For each of the $4 \times 6$ parameter combinations for $M$ and $\tau$, 50 replicate data sets were simulated. There are 50 loci in each data set, with 20 sequences from each of the three species, and 500 sites in the sequence. The data were simulated using the mccoal program, part of the bpp release, as detailed in Zhang et al. (2011). We used bpp version 4.0 to estimate the parameters in the MSC model on the fixed species tree $((A, B), C)$ (this is the A00 analysis of Yang, 2015). Version 4.0 of the program assigns inverse-gamma priors on parameters. We used the shape parameter 3 in the inverse-gamma priors, while the prior means are set to match the true values: $\theta \sim$ IG(3, 0.01) with mean $0.01/(3-1) = 0.005$, and $\tau_{ABC} \sim $ IG(3, 0.025), IG(3, 0.05), and IG(3, 0.1), for the three true $\tau_{ABC}$ values. Note that the value 3 for the shape parameter means that the inverse-gamma priors are diffuse, with the coefficient of variation to be $1/\sqrt{\alpha - 2} = 1$. Estimation of parameters under the MSC is known to be fairly robust to the priors, for example, to a one order-of-magnitude change to the prior means (Burgess and Yang 2008). After bpp generated the posterior distribution of the parameters, we used the posterior means to calculate *gdi* using equation 7, with $\tau = \tau_{AB}$ and $\theta = (\theta_A + \theta_B)/2 $.

**Figure 4.**
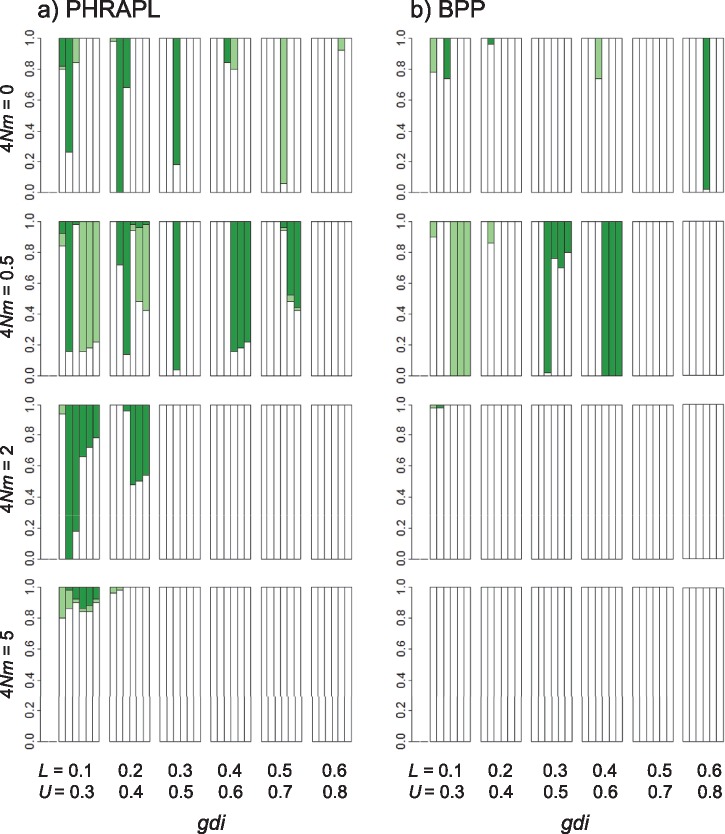
Accuracy of species delimitation using the *gdi* with parameters estimated from data of 50 loci using (a) phrapl and (b) bpp. Species status is defined using the *gdi* at different cutoffs ($L$ and $U$). This is calculated by simulating 10,000 gene trees under the MSC model with migration for phrapl, and analytically for bpp. Along the $x$-axis, each group of bars gives results for different *gdi* cut-offs. Below the lower bound ($L$), populations $A$ and $B$ are defined as a single species; above the upper bound ($U$), $A$ and $B$ are defined as separate species, while between the bounds, the species status is ambiguous. The six bars within each group represent the six sets of species divergence times ($\tau$s). The bar shadings are white = the inferred delimitation outcome matched the true outcome; light green/gray = ambiguity was inferred when the true delimitation is known (insufficient power); dark green/gray = delimitation was inferred (whether one or two species) when the truth was ambiguous (excessive confidence); and black = one species was inferred when there were two, or vice versa. The results for phrapl are recreated using the R code from Jackson et al. (2017, Fig. 4).

The results are shown in Figure 4. Even though it ignores migration and uses an overly simplistic JC mutation model (while the true model is HKY), bpp performed better than phrapl in delimiting species status defined by the *gdi*, especially at high migration rates (with $4Nm$ = 2 or 5). This result may seem counterintuitive, since the data were simulated with migration and phrapl allows for migration so that there is no model violation, while bpp ignores migration so that its model is violated.

### Shortcomings of Approximate Methods

We suggest that two factors may account for the poorer performance of phrapl in this simulation. First, phrapl is a summary method for estimating parameters, and it relies on gene tree topologies and ignores branch lengths. As a result, parameter estimates may be biased or even inconsistent due to phylogenetic errors of gene tree reconstruction (Yang 2002). Second, use of the gene tree topologies, while ignoring the branch lengths leads to information loss and may even cause identifiability problems. In the simple case of three species and three sequences, with one sequence from each species, there is only one degree of freedom in the data of gene tree topologies, which is the proportion of the most common gene tree topology. In this case the complete-isolation model (with $M = 0$) involves four parameters (two $\tau$s and two $\theta$s for the two ancestral species), but use of the gene tree topologies alone allows the estimation of only the internal branch length on the species tree in coalescent units, $2 (\tau_{ABC} - \tau_{AB}) / \theta_{AB}$, while other parameters are unidentifiable (Xu and Yang 2016). Even the internal branch length is estimated inconsistently because phylogenetic reconstruction errors tend to inflate gene tree-species tree mismatches (Yang 2002).

The cases with more than three sequences per locus and with migration may be more complex, but it should not be surprising that approximate methods that rely on summary statistics such as gene tree topologies will suffer from an information loss. In contrast, bpp is a full-likelihood method and makes use of information in the gene tree branch lengths (coalescent times) as well as topologies, while accommodating phylogenetic uncertainties due to the limited number of informative sites at each locus (Yang 2014; Xu and Yang 2016). Even though bpp operates under a wrong model that ignores migration, the sequence data at multiple loci may be informative about the expected gene tree configurations. Nevertheless, extension of bpp to allow for gene flow will provide more accurate estimation of parameters in the MSC model, which should lead to more accurate species delimitation using heuristic criteria such as *gdi*.

## Heuristic Species Delimitation Using bpp

Here, we describe how Bayesian parameter estimation under the MSC model can be combined with *gdi* to delimit species using a hierarchical procedure based on a species/population tree. This is similar to the use of a “guide tree” for species delimitation by Yang and Rannala (2010), in that an ancestral node on the guide tree is merged into one species only if its descendant nodes are merged. However, here, we rely on Bayesian parameter estimation on a fixed species/population tree while Yang and Rannala (2010) used reversible-jump algorithms to calculate posterior probabilities for different species delimitation models (represented by merging nodes on the guide tree). We first demonstrate the procedure using a simulated data set and then apply it to the analysis of three empirical data sets analyzed previously by Jackson et al. (2017). The *gdi* is only one of many possible heuristics with rough correspondences to different species definitions.

**Figure 5.**
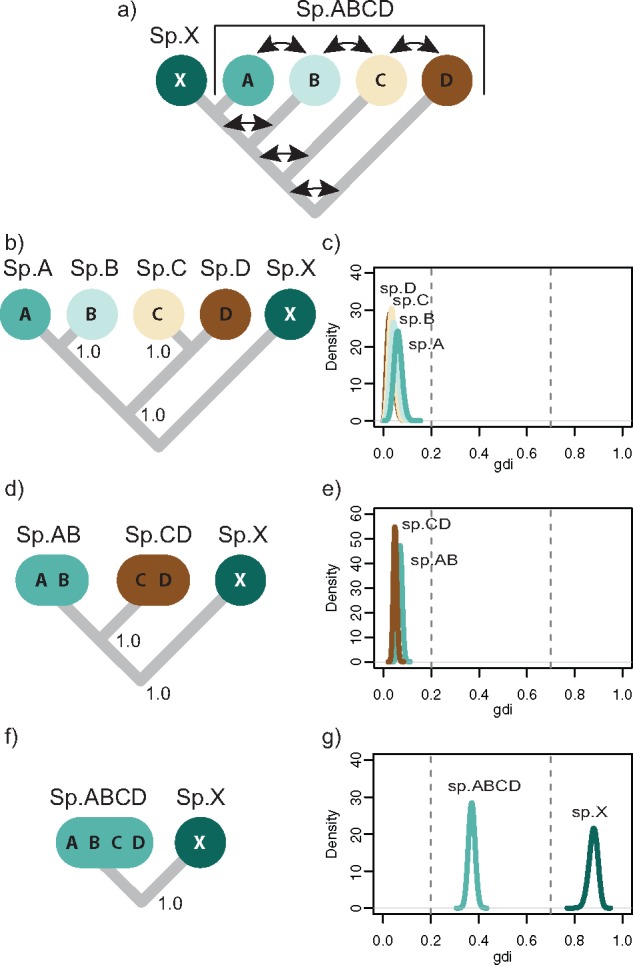
Species delimitation applying heuristic index *gdi* to parameter estimates from bpp. a) Species tree used for simulation allows migration between populations $A, B, C$, and $D$ and their ancestors (indicated by arrows), but no gene flow involving species $X$. b) Species (guide) tree inferred from A11 analysis of bpp. In (b–g), *gdi* is used to collapse populations on guide tree into same species in a hierarchical procedure, with bpp used to estimate MSC parameters ($\theta$ and $\tau$) and generate posterior distribution of *gdi*. For example, *gdi* calculated using population $A$ of panel b, based on $2\tau_{AB}/\theta_A$ (equation 7), is shown in panel c (labeled ‘sp. $A$’). Sister populations inferred to belong to same species by *gdi* are collapsed, and resulting species tree is used to conduct a new bpp analysis. Procedure is repeated until distinct species are inferred or until root of tree is reached. According to Jackson et al. (2017), *gdi*$ < 0.2$ indicates a single species, *gdi*$ > 0.7$ indicates distinct species, and *gdi* values between 0.2 and 0.7 represent ambiguous species status.

We use a species/population tree for five populations, $((((X, A), B), C), D)$, to simulate data (Fig. 5a). $ABCD$ represents a large paraphyletic species with a broad geographic distribution arranged in a stepping-stone design, with migration between any two adjacent populations including the ancestors (e.g., between $D$ and the ancestral population $XABC$ after the first population split, and then between $C$ and $D$ and between $C$ and $XAB$ after the second split, etc.). The scaled migration rate is $M = Nm = 2$ for any pair of adjacent populations. $X$ is a new species, having separated from population $A$ (with $\tau_{XA} = 0.01$), and there is no gene flow involving $X$. The divergence times ($\tau$s) are at 0.04, 0.03, 0.02, and 0.01. The population size parameter is $\theta = 0.01$ for all populations. We simulated 100 loci, each of 500 sites, for four samples per species (20 sequences per locus).

To generate a working species/population tree (the guide tree), we run a joint analysis of species delimitation and species tree estimation (the A11 analysis in bpp, Yang, 2015). The parameters in the MSC model are assigned diffuse inverse-gamma priors $\theta \sim \textrm{IG}(3, 0.02)$ and $\tau \sim \textrm{IG}(3, 0.08)$, with shape parameter 3 and with the prior means matching the true values. We used a burnin of 40,000, sample frequency of 10, and collected 50,000 samples. We conducted four separate runs for each analysis, with convergence ensured mainly by checking consistency between runs. The posterior probabilities for the species delimitation models calculated in the A11 analysis provided strong support for five species, and the inferred species tree incorrectly placed species $X$ sister to $ABCD$ (Fig. 5b). This incorrect topology may be expected, as populations exchanging genes tend to form clades in species tree analyses that ignore migration (Leaché et al., 2013). Next, we run an A00 analysis, estimating parameters on the inferred guide tree (Fig. 5b) to generate the posterior distribution for the *gdi* for the most recent species divergences, between $A$ and $B$ and between $C$ and $D$ (Fig. 5c). Note that $2\tau_{AB}/\theta_A$ is used to decide whether population $A$ is a species distinct from $B$, while $2\tau_{AB}/\theta_B$ is used to decide whether population $B$ is a species distinct from $A$. Low *gdi* values of $< 0.2$ indicate that $A$ and $B$ are one species, as are $C$ and $D$. Next, we collapse $A$ and $B$, and $C$ and $D$, and conduct another A00 analysis to estimate $\theta$ and $\tau$ for putative species $AB$ and $CD$ (Fig. 5d). The posterior distribution of *gdi* obtained suggest that $AB$ and $CD$ belong to the same species (Fig. 5e). The final iteration fits a two-species model containing species $X$ and species $ABCD$ (Fig. 5f). The *gdi* value for species $ABCD$ is ambiguous (with $0.2 <$*gdi*$< 0.7$), while the evidence for species $X$ is strong (*gdi*$>0.7$, Fig. 5g). Here, the *gdi* shows an ambiguity of the species status of $X$ and $ABCD$, depending on which population size ($\theta_X$ or $\theta_{ABCD}$) is used to calculate the index.

Next, we re-analyzed the three empirical data sets of Jackson et al. (2017) using the hierarchical procedure described above. The three empirical data sets include eight nuclear loci from three populations of North American ground skinks (*Scincella lateralis*), 20 loci from three populations of southeastern United States pitcher plants (*Sarracenia alata*), and 50 loci from four population of *Homo sapiens*. In the analysis of Jackson et al. (2017), phrapl supported a single species of *Scincella lateralis* and two species of *Sarracenia alata*, and grouped the human populations into one species, while Bayesian model selection by bpp inferred the maximum number of species in each data set.

**Figure 6.**
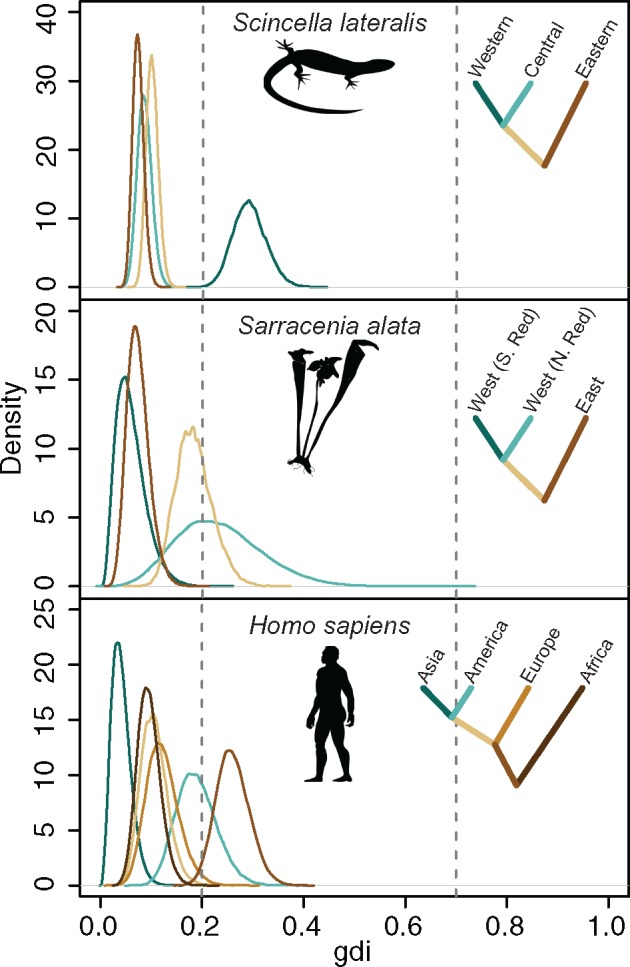
Posterior distribution of genealogical divergence index (*gdi*), generated in bpp analysis of three real data sets of Jackson et al. (2017). Silhouettes of species are from phylopic.org http://phylopic.org. Colored ancestral branches were analyzed by collapsing descendent species and conducting new MCMC analyses.

Here, we used the MCMC samples generated in the bpp analysis (Yang, 2015, analysis A00) to estimate the posterior distribution of the *gdi*. We used inverse-gamma priors on parameters ($\theta$s and $\tau$s), with the shape parameter 3 and with the same prior means as used by Jackson et al. (2017). For each data set, we conducted four separate runs with a burnin of 10,000, sample frequency of 5, and collected 100,000 samples. The guide species trees are fixed at the previously published topologies from Jackson et al. (2017) (Fig. 6). We applied the hierarchical procedure to calculate *gdi* for population pairs by collapsing populations into a single species and conducting new MCMC analyses. Using bpp to calculate posterior distributions for *gdi*, we find no support for multiple species (*gdi*$> 0.7$) in any of the empirical data sets (Fig. 6).

## Discussion

### Simulation of Species Divergences

The PSM specifies a process of population splits (incipient species formation) as well as conversions of incipient species (populations) into true species. However, with time running forward, simulation under the PSM produces a new species (a conversion event) instantaneously. At a conversion event, the new true species and its parental incipient species (population) are deemed distinct species. As stated above, this process does not realistically model the biological process of speciation, nor does it mimic the way taxonomists identify new species. We consider two alternative approaches for simulating the process of population splits and species assignments, and discuss their implications for the development of methods for species delimitation using genomic sequence data. A clear specification of the simulation procedure implies a probabilistic model of data generation and statistical inference methodology, because given the model, full-likelihood methods (maximum likelihood and Bayesian inference) are known to have certain desirable statistical properties (Rannala 2015).

In the first approach, one can simulate population splits under a branching model, such as the birth–death process. The random birth and death events specify a probabilistic distribution of the population tree topology and divergence times ($\tau$s), and a certain model may be used to sample the population sizes ($\theta$s) and migration rates ($M$s). Gene trees (topologies and coalescent times) can be generated using the population tree with parameters ($\tau$s, $\theta$s, $M$s), and then used to simulate sequence alignments. At the end of this simulation, the populations at the tips of the population phylogeny are assigned species status using heuristic criteria of divergence times and migration rates. This is very similar to the simulation approach of Jackson et al. (2017).

In the second approach, one may simulate population splits as in the first approach, but in addition simulate the evolution of a continuous character along the branches of the generated population phylogeny. The difference in the continuous character between two populations is a measure of genetic incompatibility and a threshold can be used to identify species status: if the continuous character has measurements $x_i$ and $x_j$ in two populations, they are considered distinct species if and only if $|x_i - x_j| > d$. Evolution of the continuous character may be simulated based under a model for the accumulation of genetic incompatibilities (such as the Dobzhansky–Muller incompatibilities, Orr and Turelli, 2001), for example, with a small probability for “catastrophes” (mimicking large events that may establish reproductive isolation at an instance, such as chromosomal rearrangements or polyploidizations) and a large probability for Brownian motion-like drift over time (mimicking the accumulation of genetic incompatibilities over time). At the end of the simulation, species status is assigned for populations at the tips of the tree based on the differences in the continuous character.

In both approaches, we assume that the process of sequence evolution is independent of population split events, and of the evolution of the continuous character, as expected if the neutral genome is used for species delimitation. Both scenarios seem to suggest that the only inference possible using the neutral genome is the population history and the population divergence parameters ($\theta$s, $\tau$s, and $M$s). Assignment of species status will then depend on our empirical knowledge about the level of genetic divergence between good species, or the expected amount of genetic incompatibility that may be accumulated over a given time period. Both approaches of simulation posit a protracted process of speciation (to allow accumulation of genetic incompatibilities or of differences in the continuous character), in contrast to the PSM, which assumes instantaneous speciation completed over one generation.

### Hypothesis Testing Versus Parameter Estimation and the Functionalities of bpp

The MSC model was developed for comparative analysis of the ‘neutral’ genome to estimate parameters that characterize the history of population divergences, under the assumption that natural selection has not significantly altered the genealogical histories of genomic regions (gene tree topologies and coalescent times). The MSC model does not aim to identify speciation genes or genes responsible for establishing reproductive barriers (which may be under species-specific directional selection), even though identifying such genes, however, rare they are, may greatly enrich our understanding of the origin and maintenance of species. For example, proteins involved in female and male reproduction are well-known to evolve at accelerated rates, apparently driven by natural selection due to ecological adaptations and sexual selection maintaining species boundaries (Swanson and Vacquier 2002). In a few cases where the MSC model was applied to exons or the coding genome, it was noted to produce results highly consistent with the non-coding regions of the genome (Ebersberger et al. 2007; Dalquen et al. 2017; Shi and Yang 2018). This is apparently due to the fact that most protein-coding genes are performing the same conserved functions in closely related species so that the effect of purifying selection removing nonsynonymous mutations is predominantly a reduction of the neutral mutation rate. At any rate, the MSC model treats genomic regions as neutral markers to extract information concerning genealogical histories of the populations, reflected in population divergence parameters, such as population sizes, divergence times, and migration rates.

We take it for granted that the neutral genome contains useful information about the population divergence history and about species status. In clear-cut cases, population divergence parameters should be sufficient to determine species status. For example, distantly related species can be reliably identified using a simple genetic distance threshold as in DNA-barcoding analysis (Hebert et al. 2004). The difficulty is in identifying the species boundary (the so-called boundary conditions, Moritz and Cicero, 2004) for allopatric populations with low levels of genetic divergence and possibly frequent gene flow. The definitions of races, subspecies and species are often subjective, and the neutral genome may not provide unambiguous resolution of species status (Rannala, 2015). If species divergence is due to very few genes (in the so-called speciation islands), while the rest of the genome is homogenized due to widespread interbreeding, the divergence between species may be similar to the polymorphism within species (Nadeau et al., 2012). In such cases the neutral genome may not be highly informative about the species status and use of other kinds of data, such as evidence of reproductive isolation and ecological adaptation or identification of speciation genes, may be necessary to determine species status.

The inherent subjectivity of allopatric species delimitation is clearly illustrated by the distinction between *statistical significance* and *biological significance* made by Jackson et al. (2017). Consider by analogy a coin-tossing experiment to determine whether a coin is biased. One can use a significance test to test the null hypothesis of a fair coin (with the probability of heads $p = \frac{1}{2}$) against the alternative hypothesis of a biased coin (with $p \ne \frac{1}{2}$) or calculate the posterior probabilities for the two models. With a large number of coin tosses, this approach of model selection may have the power to detect a very small bias, with $p = 0.51$, say. However, the bias of 0.01 is said to be statistically significant but not biologically significant, and it is considered incorrect to suggest that the coin with $p = 0.51$ is biased. An alternative approach is to estimate the probability parameter $p$ using the counts of heads and tails, and then apply whatever definition of bias one assumes heuristically. Given the arbitrariness in the definition of a biased coin, this approach may be the only one feasible.

Similarly, we have in this article made a distinction between two kinds of analysis under the MSC model implemented in bpp: (i) Bayesian model selection to calculate posterior probabilities for different species delimitation models (the A10 and A11 analyses in Yang, 2015) and (ii) Bayesian parameter estimation when species/population assignment and phylogeny are fixed (the A00 analysis in Yang, 2015). In theory, selection of species delimitation models can also be conducted in a Frequentist framework using a likelihood ratio test, for example, with the one-species model formulated as the null hypothesis (with $\tau =0$) and the two-species model the alternative (with $\tau > 0$). With genomic data, model selection in both the Frequentist and Bayesian frameworks may be very powerful in identifying population splits even if the age of the divergence event ($\tau$) is very young.

We suggest that Bayesian model selection is appropriate for identifying morphologically cryptic species. Even if the genomic data or the bpp program cannot distinguish populations and species, the genetic distinctness of the populations signifies the presence of reproductive barriers or isolation mechanisms. There seems to be no controversy in assigning species status to populations that exist in sympatry and are genetically distinct.

For heuristic delimitation of allopatric species, we suggest the use of Bayesian parameter estimation. The genomic data allows reliable estimation of population-divergence parameters ($\theta$s, $\tau$s, and $M$s), which can then be used to apply a heuristic definition of species status.

### Heuristic Criteria for Species Status

The *gdi* attempts to use the overall genetic divergence between two populations affected by the combined effects of genetic isolation and gene flow. The index appears to have weaknesses. First, the criterion depends on the population divergence time relative to the population size ($2\tau/\theta_A$ in the case of no gene flow). If the population is established by a few founder individuals, $N_A$ and $\theta_A$ may be very small, and the use of *gdi* may lead to claims of species status even if the populations diverged very recently. It may be necessary to consider the (absolute) population divergence ($\tau$) (Yang and Rannala, 2010) as well as the divergence relative to the population size. Second, there may be ambiguity when the two populations concerned have very different sizes. If $N_A \ll N_B$, the use of *gdi* may lead to the awkward solution that $A$ is a distinct species from $B$ (if one uses sequences $a_1, a_2$ and $b$ to calculate the index) but $B$ is not a distinct species from $A$ (if one uses sequences $a$, $b_1, b_2$). This is the case in the analysis of the simulated data in Fig. 5g. Third, *gdi* has a large range of indecision (0.2–0.7), although this may reflect the arbitrary nature of species definition rather than a weakness of the index itself.

There is clearly a need to refine criteria for heuristic species delimitation using genomic sequence data. It may be necessary to incorporate multiple criteria. For example, we may require a minimum species divergence time relative to the population size ($2\tau/\theta > 1$), a minimum absolute divergence time ($> 10^4$ generations, say, as indicated by $\tau$), and a maximum migration rate between species ($M = Nm < 1$). If a contact zone exists for the two populations, important indicators of pre- and post-mating reproductive isolation may be obtainable. For example, we may require the frequency of F$_1$ hybrids ($f$) to be $< 10\%$ of the frequency expected from population abundance, and we may further require the long-term migration rate to be much lower than the hybrid frequency (with $m < 0.1f$, say), indicating selective rejection of introgressed alleles after hybridization.

### Concluding Remarks

The MSC model and its implementation in bpp provides a powerful framework for inferring population divergence histories and estimating evolutionary parameters using the fast-accumulating genomic sequence data. There appears to be no controversy regarding the use of Bayesian model selection under MSC or bpp to identify morphologically cryptic species. For allopatric populations or species, the accurate estimation of important population parameters should allow one to apply any empirical criterion for defining species that the evolutionary biologist entertains. For these reasons, the MSC model and bpp will continue to be useful tools in the analysis of genomic data to better understand biodiversity despite the fact that the interpretation of these results in assessing species status may be debated.

## Supplementary Material

Data available from the Dryad Data Repository: http://dx.doi.org/10.5061/dryad.t66gq81.
